# Effects of plasma concentrations of 5-fluorouracil on long-term survival after treatment with a definitive 5-fluorouracil/cisplatin-based chemoradiotherapy in Japanese patients with esophageal squamous cell carcinoma

**DOI:** 10.1186/1756-9966-30-94

**Published:** 2011-10-05

**Authors:** Akiko Kuwahara, Motohiro Yamamori, Kaori Kadoyama, Kohshi Nishiguchi, Tsutomu Nakamura, Ikuya Miki, Takao Tamura, Tatsuya Okuno, Hideaki Omatsu, Toshiyuki Sakaeda

**Affiliations:** 1 School of Pharmacy and Pharmaceutical Sciences, Mukogawa Women's University, Nishinomiya 663-8179, Japan; 2 Kobe University Graduate School of Medicine, Kobe 650-0017, Japan; 3 Graduate School of Pharmaceutical Sciences, Kyoto University, Kyoto 606-8501, Japan; 4 Faculty of Pharmaceutical Sciences, Kyoto Pharmaceutical University, Kyoto 607-8414, Japan

**Keywords:** esophageal squamous cell carcinoma, 5-fluorouracil, plasma concentration, clinical outcome, prognosis

## Abstract

**Background:**

A substantial body of literature has accumulated during the past 20 years showing the plasma concentrations of 5-fluorouracil (5-FU) to correlate with clinical response and/or toxicity in colorectal cancer, and head and neck cancer, but little information is available concerning effects on long-term survival. Here, Japanese patients with esophageal squamous cell carcinoma (ESCC) were followed up for 5 years after treatment with a definitive 5-FU/cisplatin (CDDP)-based chemoradiotherapy (CRT), and the association between prognosis and the plasma concentration of 5-FU was evaluated.

**Methods:**

Forty-nine patients with ESCC, who were treated with a definitive 5-FU/CDDP-based CRT, were enrolled. A course consisted of the continuous infusion of 5-FU at 400 mg/m^2^/day for days 1-5 and 8-12, the infusion of CDDP at 40 mg/m^2^/day on days 1 and 8, and the radiation at 2 Gy/day on days 1 to 5, 8 to 12, and 15 to 19, with a second course repeated after a 2-week interval. Plasma concentrations of 5-FU were determined by high performance liquid chromatography at 5:00 PM on days 3, 10, 38 and 45, and at 5:00 AM on days 4, 11, 39 and 46.

**Results:**

The overall 5-year survival rate was 42.9%. Age (P = 0.020), body weight (P = 0.019), and disease stage (P = 0.048) affected the survival, and the survival depended on the clinical response assessed at 1 month after the treatment (P = 0.001). Higher plasma concentrations of 5-FU resulted in a better clinical response (P = 0.043), and trended to prolong survival (P = 0.321).

**Conclusions:**

The long-term survival after treatment with a definitive 5-FU/CDDP-based CRT possibly depends on the plasma concentrations of 5-FU, and further clinical studies with a larger number of cases are needed to clarify the relationship between them.

## Background

A clinical report published in 1999, the RTOG (Radiation Therapy Oncology Group) 85-01 trial involving 134 patients with T1-3, N0-1 and M0 esophageal cancer, is of great interest in terms of clinical outcome because it demonstrated a 5-year survival rate of 26% [1-4]. This treatment consists of a 96-hr-infusion of 5-fluorouracil (5-FU) at a daily dose of 1,000 mg/m^2^/day in weeks 1, 5, 8 and 11, infusion of cisplatin (CDDP) at 75 mg/m^2^/day on the first day of weeks 1, 5, 8 and 11, and concurrent radiation at 50 Gy in 25 fractions over 5 weeks, without pre- or post-surgical resection. Simultaneously in Japan, another version was proposed by Ohtsu and his co-workers for advanced metastatic esophageal squamous cell carcinoma (ESCC) which consists of a 120-hr-infusion of 5-FU at 400 mg/m^2^/day in weeks 1, 2, 6 and 7, infusion of CDDP at 40 mg/m^2^/day on the first day of weeks 1, 2, 6 and 7, and concurrent radiation at 60 Gy in 30 fractions over 8 weeks [5,6]. Two independent clinical investigations have shown curative potential using this regimen for unresectable ESCC with T4 or M1a [5,6], and a long-term evaluation of efficacy and toxicity with 139 patients resulted in a complete response (CR) rate of 56%, along with a 5-year survival rate of 29% [7-9]. Currently, a definitive 5-FU/CDDP-based chemoradiotherapy (CRT) is recognized as one of the most promising treatments for esophageal cancer, but given the extensive inter-individual variation in clinical outcome and severe late toxicities, future improvements will likely require the dose-modification of these regimens, incorporation of a novel anticancer drug, pharmacokinetically guided administration of 5-FU or CDDP, and identification of responders via patient genetic profiling [10].

5-FU exerts its anticancer effects through inhibition of thymidylate synthase and incorporation of its metabolites into RNA and DNA, and has been used widely for the treatment of solid tumors for nearly 50 years [11]. A substantial body of literature has accumulated over the past 20 years showing the plasma concentrations of 5-FU to correlate with clinical response and/or toxicity in colorectal cancer, and head and neck cancer [12-21]. Although the therapeutic drug monitoring has not been used for chemotherapeutic agents [22,23], the accumulation of data has encouraged us to apply this strategy in the case of 5-FU [24,25]. There are only 2 reports in which plasma concentrations of 5-FU has been shown to correlate with long-term survival [16,18], but Gamelin and his co-workers conducted a phase III, multicenter, randomized trial in which pharmacokinetically guided administration of 5-FU was compared with conventional dosing in patients with metastatic colorectal cancer, and concluded that individual dose adjustments of 5-FU resulted in an improved objective response rate and fewer severe toxicities, and in a trend toward a higher survival rate [21].

A series of studies has been performed to find a marker predictive of clinical response 1 month after or severe toxicities during treatment with a definitive 5-FU/CDDP-based CRT in Japanese patients with ESCC [26-31]. Obviously, the final goal of cancer chemotherapy is an improvement in long-term survival, not a short-term clinical response, so parameters predicting prognosis have been absolutely imperative. In this study, patients with ESCC were followed up for 5 years after treatment with a definitive 5-FU/CDDP-based CRT. This is the first report on the effects of plasma concentrations of 5-FU on long-term survival in cases of esophageal cancer.

## Methods

### Patients

Forty-nine ESCC patients were enrolled in this study based on the following criteria: 1) ESCC treated with a definitive 5-FU/CDDP-based chemoradiotherapy at Kobe University Hospital, Japan, from October, 2003 to June, 2006; 2) clinical stage T1 to T4, N0 or N1, and M0 or M1a according to the International Union Against Cancer tumor-node-metastasis (TNM) classification; 3) age less than 85 years; 4) an Eastern Cooperative Oncology Group performance status of 0 to 2; 5) adequate bone marrow, renal, and hepatic function; 6) no prior chemotherapy; 7) no severe medical complications; and 8) no other active malignancies (except early cancer). The tumors were histologically confirmed to be primary, and no patients with recurrence were included in this study.

### Protocol

**Figure 1:**
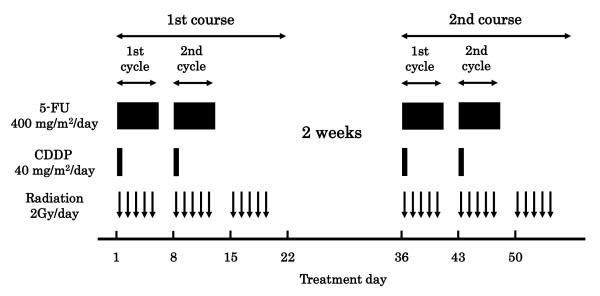
**Protocol of a definitive 5-fluorouracil/cisplatin-based chemoradiotherapy**. One course of treatment consisted of protracted venous infusions of 5-fluorouracil (400 mg/m^2^/day for days 1-5 and 8-12) and cisplatin (40 mg/m^2^/day on days 1 and 8), and radiation (2 Gy/day on days 1-5, 8-12, and 15-19), with a second course (days 36-56) repeated after a 2-week interval.

The protocol is presented in Figure 1. A course consisted of the continuous infusion of 5-FU at 400 mg/m^2^/day for days 1-5 and 8-12, the infusion of CDDP at 40 mg/m^2^/day on days 1 and 8, and the radiation at 2 Gy/day on days 1 to 5, 8 to 12, and 15 to 19, with a second course repeated after a 2-week interval [5,6]. If disease progression/recurrence was observed, either salvage surgery, endoscopic treatment, or another regimen of chemotherapy was scheduled. This study was conducted with the authorization of the institutional review board and followed the medical research council guidelines of Kobe University. Written informed consent was obtained from all participants prior to enrollment.

### Determination of plasma concentrations of 5-FU

Aliquots (5 mL) of blood were collected into etylenediaminetetraacetic acid-treated tubes at 5:00 PM on days 3, 10, 38, and 45, and at 5:00 AM on days 4, 11, 39, and 46 [26-30]. The plasma concentrations of 5-FU were determined by high-performance liquid chromatography as described previously [26-30].

### Clinical response

The clinical response was evaluated as reported previously [5-9]. Briefly, a complete response (CR) was defined as the complete disappearance of all measurable and assessable disease at the first evaluation, which was performed 1 month after the completion of CRT to determine whether the disease had progressed. The clinical response was evaluated by endoscopy and chest and abdominal computed tomography (CT) scans in each course. A CR at the primary site was evaluated by endoscopic examination when all of the following criteria were satisfied on observation of the entire esophagus: 1) disappearance of the tumor lesion; 2) disappearance of ulceration (slough); and 3) absence of cancer cells in biopsy specimens. If small nodes of 1 cm or less were detected on CT scans, the recovery was defined as an "uncertain CR" after confirmation of no progression for at least 3 months. An "uncertain CR" was included as a CR when calculating the CR rate. When these criteria were not satisfied, a non-CR was assigned. The existence of erosion, a granular protruded lesion, an ulcer scar, and 1.2 w/v% iodine/glycerin-voiding lesions did not prevent an evaluation of CR. The evaluations were performed every month for the first 3 months, and when the criteria for CR were not satisfied at 3 months, the result was changed to non-CR. Follow-up evaluations were performed thereafter every 3 months for 3 years by endoscopy and CT scan. After 3 years, patients were seen every 6 months. During the follow-up period, a routine course of physical examinations and clinical laboratory tests was performed to check the patient's health.

### Severe acute toxicities

A definitive 5-FU/CDDP-based CRT is associated with acute toxicities, predominantly leucopenia, stomatitis, and cheilitis [5-9]. Toxicity was evaluated using criteria defined by the Japan Clinical Oncology Group [32]. These criteria were based on the National Cancer Institute Common Toxicity Criteria. Toxicity was assessed on a 2 to 3 day basis during the CRT and subsequent hospitalization period and on every visit after the completion of CRT. Episodes of leucopenia, stomatitis, and cheilitis during the first 2 courses and subsequent 2 weeks (until day 70) were recorded as acute toxicities and those of grade 3 or more as severe acute toxicities.

### Survival after treatment with a 5-FU/CDDP-based CRT

Survival time was defined as the time from treatment initiation to death from any cause or to the last date of confirmation of survival. Survival data were updated on June 25, 2011.

### Data analysis and statistics

All values reported are the mean ± standard deviation (SD). The unpaired Student's *t*-test/Welch's test or Mann-Whitney's *U *test was used for two-group comparisons, and AVOVA was for multiple comparisons. Fisher's exact test was also used for the analysis of contingency tables. The difference of overall survival curves was analyzed by Log-rank test. P values of less than 0.05 (two tailed) were considered to be significant.

## Results

Demographic/clinicopathologic characteristics and clinical outcome of 49 Japanese ESCC patients are summarized in Table 1. The 1-year, 2-year, and 5-year survival rates were 71.4%, 57.1%, and 42.9%, respectively. The patients who survived 5 years or more were older (P = 0.020) and heavier (P = 0.019) than those who lasted less than 5 years. There was a significant difference in disease stage between the 2 groups (P = 0.048). The CR rate was 76.2% for the patients surviving 5 years or more, but only 25.0% for the others (P = 0.0005). No differences were found in the frequency of episodes of severe acute leucopenia, stomatitis, and cheilitis.

**Table 1:** Demographic/clinicopathologic characteristics and clinical outcome after treatment with a definitive 5-fluorouracil/cisplatin-based chemoradiotherapy in 49 Japanese patients with esophageal squamous cell carcinoma

Group	Total	Survival of 5 years or more	Survival of less than 5 years	P ^a)^
N	49	21	28	
1) Demographic/clinicopathologic				
Age, yr	64.5 ± 7.4 (48 -78) ^b)^	67.3 ± 5.8 (60 -78)	62.4 ± 7.9 (48 -76)	0.020
Height, cm	163.5 ± 6.6 (150-180)	161.9 ± 6.1 (150-171)	164.8 ± 6.8 (152-180)	0.125
Weight, kg	56.1 ± 9.6 (33-79)	59.8 ± 9.5 (40-74)	53.3 ± 8.9 (33-79)	0.019
Male/Female	46/3	20/1	26/2	1.000
Performance status, 0/1/2/unknown	24/20/4/1	11/7/2/1	13/13/2/0	0.579
Differentiation, well/moderate/poor/unknown	7/28/8/6	4/11/3/3	3/17/5/3	0.817
T1/T2/T3/T4	16/6/15/12	10/2/7/2	6/4/8/10	0.099
N0/N1	22/27	13/8	9/19	0.048
M0/M1a ^c)^	41/8	20/1	21/7	0.115
Stage I/II/III/IV	12/10/19/8	7/7/6/1	5/3/13/7	0.048
				
2) Clinical outcome				
Complete response	23 (46.9%)	16 (76.2%)	7 (25.0%)	0.0005
Grade 3/4 Leucopenia	21(42.9%)	9 (42.9%)	12 (42.9%)	1.000
Grade 3/4 Stomatitis	7 (14.3%)	4 (19.0%)	3 (10.7%)	0.443
Grade 3/4 Cheilitis	8 (16.3%)	4 (19.0%)	4 (14.3%)	0.710

a) Survival of 5 years or more vs. less than 5 years.

b) The values are the mean ± SD, with the range in parentheses.

c) Noncervical primary tumors with positive supraclavicular lymph nodes were defined as M1a.

**Figure 2:**
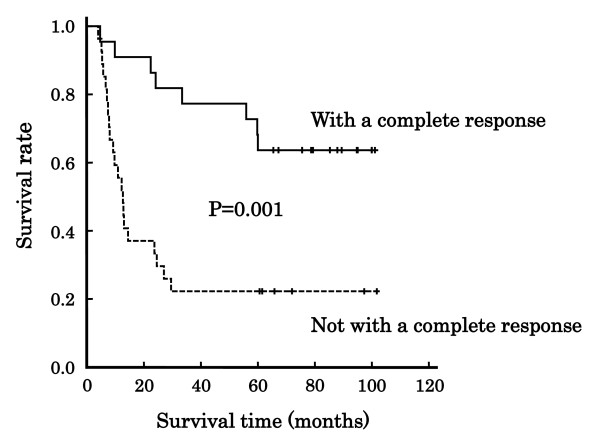
**Association of clinical response with overall survival in Japanese patients with esophageal squamous cell carcinoma**. Line: patients with a complete response (CR, N = 23), dotted line: patients not with a complete response (non-CR, N = 26). The survival depended on the response (P = 0.001, Log-rank test).

**Figure 3:**
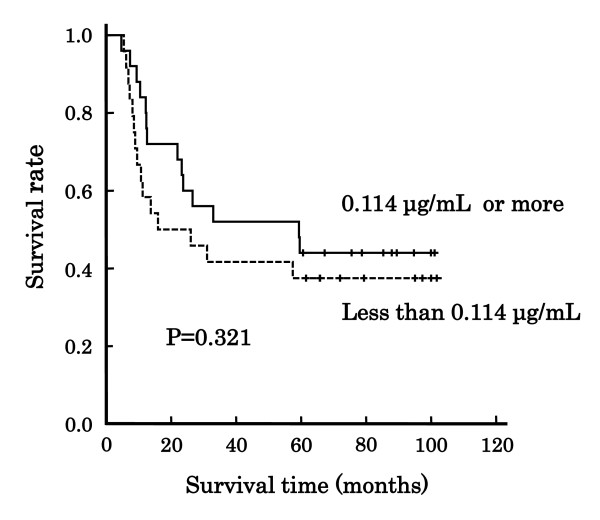
**Association of 8-point average of plasma concentrations of 5-fluorouracil with overall survival in Japanese patients with esophageal squamous cell carcinoma**. Line: patients with plasma concentrations of 5-FU of 0.114 μg/mL or more (N = 25), dotted line: patients with plasma concentration of 5-FU of less than 0.114 μg/mL (N = 24). No statistical significant difference was observed (P = 0.321, Log-rank test).

Figure 2 shows the association of clinical response with overall survival after the treatment with a definitive 5-FU/CDDP-based CRT in 49 patients with ESCC. The survival depended on the response, i.e., CR or non-CR (P = 0.001, Log-rank test). The plasma concentrations of 5-FU in the patients with a survival time of 5 years or more and with less than 5 years are indicated in Table 2. There was no difference of the 8-point average values of plasma concentrations of 5-FU between the 2 groups (P = 0.536), although the clinical response depended on; 0.124 ± 0.036 μg/mL for CR, 0.105 ± 0.030 μg/mL for non-CR (P = 0.043). Figure 3 shows the association of the 8-point average value with overall survival. The patients were divided into 2 groups based on an overall average of 0.114 μg/mL, and again the effect on overall survival was not confirm (P = 0.321, Log-rank test). The plasma concentrations of 5-FU in the patients with CR, but a survival period of less than 5 years, are listed in Table 3. The 8-point average of the concentrations tended to be higher than other subgroups (P = 0.226).

**Table 2:** Plasma concentrations of 5-fluorouracil (μg/mL) during a definitive 5-fluorouracil/cisplatin-based chemoradiotherapy in 49 Japanese patients with esophageal squamous cell carcinoma

Group		Total	Survival of 5 years or more	Survival of less than 5 years	P ^a)^
N		49	21	28	
1st cycle/1st course	Day 3, PM 5:00	0.109 ± 0.060	0.122 ± 0.080	0.100 ± 0.041	0.294
	Day 4, AM 5:00	0.076 ± 0.040	0.088 ± 0.044	0.068 ± 0.036	0.097
2nd cycle/1st course	Day 10, PM 5:00	0.150 ± 0.074	0.137 ± 0.071	0.158 ± 0.077	0.357
	Day 11, AM 5:00	0.134 ± 0.047	0.132 ± 0.048	0.136 ± 0.047	0.798
1st cycle/2nd course	Day 38, PM 5:00	0.102 ± 0.056	0.097 ± 0.067	0.105 ± 0.049	0.676
	Day 39, AM 5:00	0.076 ± 0.041	0.077 ± 0.042	0.076 ± 0.042	0.897
2nd cycle/2nd course	Day 45, PM 5:00	0.146 ± 0.080	0.158 ± 0.101	0.136 ± 0.059	0.364
	Day 46, AM 5:00	0.119 ± 0.047	0.126 ± 0.036	0.114 ± 0.054	0.399
					
Average of 8 sampling points		0.114 ± 0.034	0.118 ± 0.036	0.112 ± 0.032	0.536

a) Survival of 5 years or more vs. less than 5 years.

**Table 3:** Plasma concentrations of 5-fluorouracil (μg/mL) during a definitive 5-fluorouracil/cisplatin-based chemoradiotherapy in the patients with a complete response, but survival of less than 5 years

	Survival of 5 years or more		Survival of less than 5 years		
		
	CR ^a)^	Non-CR	CR	Non-CR	P ^b)^
N	16	5	7	21	
Average of 8 sampling points	0.122 ± 0.031	0.105 ± 0.051	0.131 ± 0.046	0.105 ± 0.024	0.226

a) Complete response

b) Assessed by ANOVA

## Discussion

Originally, 5-FU was administered alone as a bolus, but more recently, it is being administered with biomodulating agents and/or through continuous infusion [11,33]. Because of the preclinical evidence that increased exposure to 5-FU improves its cytotoxic activity and the fact that 5-FU has a short half-life in plasma, continuous infusion has been proposed to increase the percentage of tumor cells exposed to 5-FU [33]. These regimens have resulted in improvements in response rates with improved safety profiles in clinical studies [33]. At present, one of the most important factors complicating the clinical use of 5-FU is extensive inter- and/or intra-individual variability in pharmacokinetics, when doses are calculated based on body surface area [24,25]. There is a need to individualize 5-FU dosing, and the shift from a bolus to continuous infusion has created better conditions for dose management [24,25]. Given that the plasma concentration of, or systemic exposure to, 5-FU has been shown to correlate with the response rate or the rate of adverse effects in patients with advanced colorectal cancer and head and neck cancer [12-21], pharmacokinetically guided dose adjustment has attracted attention [24,25].

To our knowledge, however, there are only 2 reports in which plasma concentrations of 5-FU were proven to correlate with long-term survival [16,18]. Milano et al. examined patients with head and neck cancer [16], and Di Paolo et al. studied patients with colorectal cancer [18], and both found that the AUC values of 5-FU were significantly correlated with survival. Recently, Gamelin and his co-workers compared pharmacokinetically guided administration of 5-FU with conventional dosing in patients with metastatic colorectal cancer, and found that individual dose adjustments of 5-FU resulted in an improved objective response rate, and in a trend toward a higher survival rate [21].

In this study, we have followed up Japanese patients with ESCC for 5 years after treatment with a definitive 5-FU/CDDP-based CRT. Age (P = 0.020), body weight (P = 0.019), and disease stage (P = 0.048) affected the long-term survival, and the survival depended on the clinical response assessed at 1 month after the treatment, i.e., CR or non-CR (P = 0.001, Figure 2). The clinical response was determined by the 8-point average values of plasma concentrations of 5-FU; 0.124 ± 0.036 μg/mL for the patients with CR, and 0.105 ± 0.030 μg/mL for those with non-CR (P = 0.043), and therefore the survival must be associated with the concentrations. However, the concentrations were not high enough to affect long-term survival (P = 0.321, Figure 3). This is presumably due to low number of patients (N = 49), and further clinical studies with a larger number of cases are needed to clarify the effect on long-term survival.

A subgroup analysis suggested plasma concentrations of 5-FU to be higher in the patients with CR, but a survival period of less than 5 years, but there was no statistical significance (Table 3). Death from esophageal cancer often occurs in non-CR cases or in recurrent cases. However, the reports indicated severe late toxic effects, such as myocardial infarction, pericardial effusion, and pleural effusion, in patients after a definitive 5-FU/CDDP-based CRT, especially in cases of extensive radiation [8,9]. Here, 2-5 of 49 patients seemed to have died from late toxicity. This might affect the association of the plasma concentrations of 5-FU with long-term survival.

## Conclusions

Japanese ESCC patients were followed up for 5 years after treatment with a definitive 5-FU/CDDP-based CRT, and the association between prognosis and the plasma concentration of 5-FU was evaluated. Age, body weight, and disease stage affected the log-term survival, and the survival depended on the clinical response assessed at 1 month after the treatment. Higher plasma concentrations of 5-FU resulted in a better clinical response, and tended to prolong survival. Further clinical studies with a larger number of cases are needed to clarify the effect on long-term survival.

## Competing interests

The author declares that they have no competing interests.

## Authors' contributions

AK, TT and TS conceived, designed and coordinated the study. IM, TT, TO and HO evaluated the clinical outcome. TN and IM determined the plasma concentrations of 5-FU. AK, MY, KK and KN carried out the data management and statistical analysis. AK and TS prepared the manuscript. All authors read and approved the final manuscript.
